# Observations of the genomic landscape beyond 1p19q deletions and EGFR amplification in glioma

**DOI:** 10.1186/s13039-015-0156-1

**Published:** 2015-08-07

**Authors:** Christian N. Paxton, Leslie R. Rowe, Sarah T. South

**Affiliations:** ARUP Institute for Clinical and Experimental Pathology® ARUP Laboratories, 500 Chipeta Way, Salt Lake City, UT 84108 USA; Departments of Pathology and Pediatrics, University of Utah, Salt Lake City, UT USA

**Keywords:** Microarray, OncoScan, Glioma, Genomic landscape, Copy number change, FFPE array

## Abstract

**Background:**

With recent advancements in molecular techniques, the opportunities to gather whole genome information have increased, even in degraded samples such as FFPE tissues. As a result, a broader view of the genomic landscape of solid tumors may be explored. Whole genome copy number and loss of heterozygosity patterns can advance our understanding of mechanisms and complexity of various tumors.

**Results:**

Genome-wide alterations involving copy number changes and loss of heterozygosity were identified in 17 glioma samples with positive FISH results for 1p19q co-deletions (n = 9) or EGFR amplification (n = 8). Gliomas positive for 1p19q co-deletions did not have other frequently recurrent genomic alterations. Additional copy-number alterations were observed in individual cases, and consisted primarily of large-scale changes, including gains or losses of entire chromosomes. The genomic architecture of EGFR amplified gliomas was much more complex, with a high number of gains and losses across the genome. Recurrent alterations in EGFR amplified gliomas were both focal, such as CDKN2A homozygous deletions, and large, such as chromosome 10 loss.

**Conclusions:**

Microarray enabled a broader picture of the genomic alterations occurring in glioma cases. Gliomas with 1p19q co-deletion had a relatively quiet genome, apart from the selected co-deletion. Additional alterations in isolated cases, involved primarily larger aberrations. On the other hand, EGFR amplified cases tended to be more complex and have specific abnormalities associated with the EGFR amplification. Furthermore, 1p19q co-deletions and EGFR amplification associated copy number changes appeared to often be mutually exclusive.

## Background

Single nucleotide polymorphism (SNP) arrays allow for a whole genome view of complex copy number changes (CNCs) and loss of heterozygosity (LOH), Recent microarray developments have incorporated molecular inversion probe (MIP) technology, which requires a relatively small molecular footprint (~40 bp) and is advantageous for assessing highly cross-linked and degraded DNA samples obtained from formalin-fixed, paraffin-embedded (FFPE) tissues [1, 2]. This ability to optimally assess copy number changes in FFPE tissue is significant to studying genomic alterations in solid tumors because solid tumors have traditionally been characterized through their histology and through FFPE tissue FISH. As a result, FFPE-preserved solid tumor samples have been archived for decades, awaiting improved technologies to better analyze them. MIP arrays, therefore, provide opportunities to characterize genomic profiles from archived FFPE samples that are already available for such studies.

This study focused on assessing the genomic signatures of gliomas–brain tumors derived from the glial cells. Gliomas are the most common form of primary brain tumors comprising approximately 30 % of all brain tumors. More importantly, about 80 % of all malignant brain tumors are gliomas [3]. The World Health Organization (WHO) has classified gliomas from I-IV based on their degree of aggressiveness, with WHO class I being relatively benign and WHO class IV as the most aggressive gliomal tumors [4]. The objective of this study was to investigate the genomic changes that occur in gliomas. We performed microarray analysis on 2 types of glial tumors to identify and compare the respective genomic landscapes.

## Results and discussion

The two categories of gliomas analyzed in this study were initially identified by FISH testing and subsequently assessed for CNCs using the OncoScan® array (Affymetrix, Santa Clara, CA). Global CNCs were analyzed to identify consistent patterns across each cohort. The first group consisted of nine oliogodendriolglial tumors that had 1p19q co-deletions identified by FISH analysis. The second group contained 8 glioblastomas (GBM), WHO class IV, initially characterized by EGFR amplification, also identified by FISH.

### 1p19q Co-deleted

**Fig. 1:**
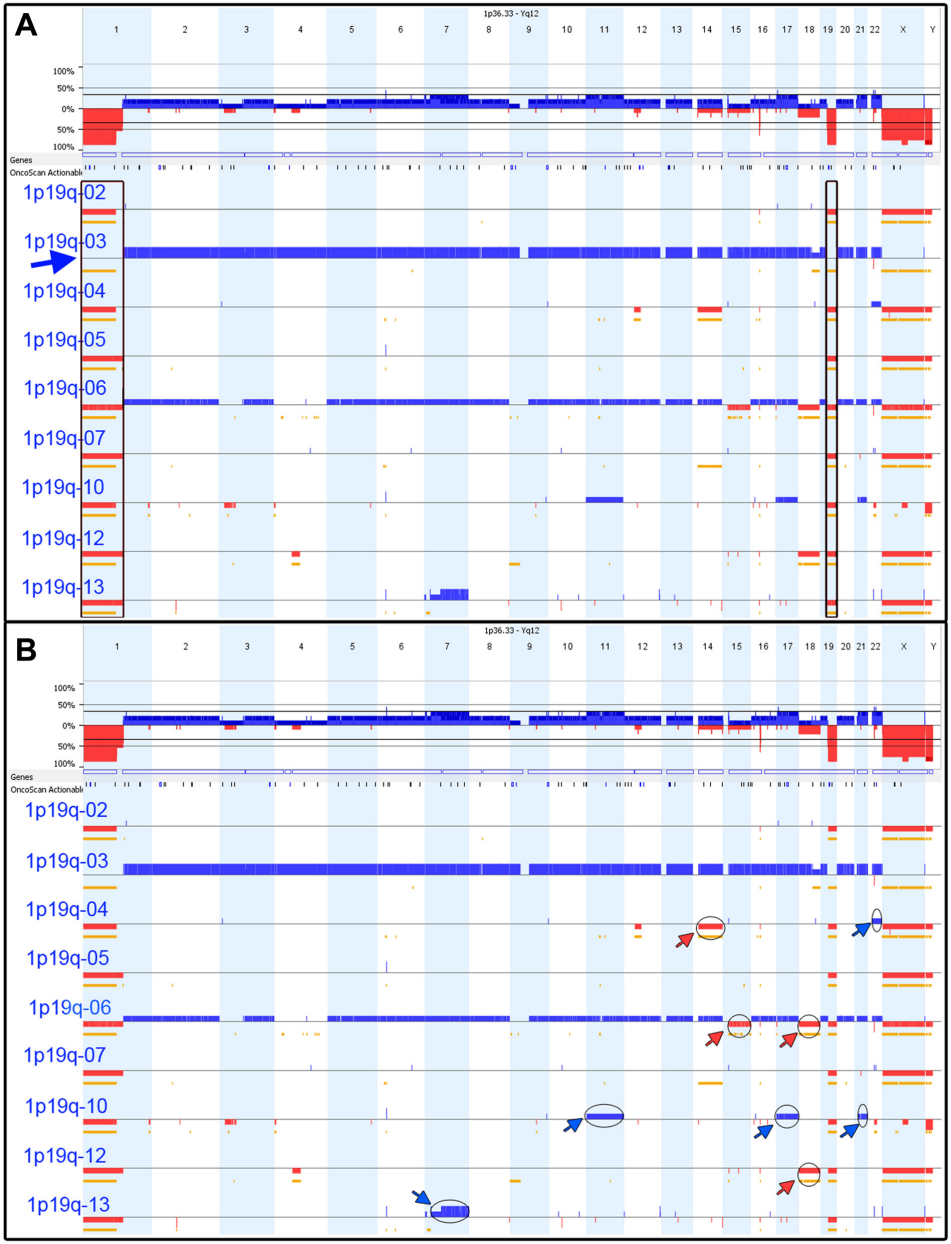
1p19q co-deletion cohort. **a** Highlights the 1p and 19q co-deletions (deletions marked by indicated region). Case 1p19q-03 had two copies of 1p and 19q, but was tetraploid throughout the remainder of the genome (blue arrow). **b** Highlights whole chromosomal changes observed in this cohort including gains (blue arrows) and deletions (red arrows)

Microarray results confirmed the 1p19q co-deletions. One strength of array technology is the broader coverage obtained in the assay. Significantly, FISH probes yield very focal results, identifying only the 200–700 kb region where the probe hybridizes. Array results show deletions of the entire 1p and 19q arms in all 9 samples (Fig. 1a). Typically, 1p19q co-deletions are consistent with a favorable prognosis, whereas partial 1p deletions are indicative of a poor prognosis [5]. Although FISH is often the first line of testing for 1p19q deletions, the technology is limited and cannot distinguish between full chromosomal arm losses as opposed to partial losses that span the probe sites.

One case (1p19q-03), with diploid copy number along 1p and 19q arms, was tetraploid across the remainder of the genome (Fig. 1a). Genotyping data from the array indicate that 1p and 19q were co-deleted prior to a doubling of the genome, so that although diploid in number, the 1p and 19q arms are still deleted in the context of the entire genome. This is consistent with the FISH findings which compare the 1p or 19q probe to a control probe found on the opposing chromosome arm.

Overall the 1p19q cohort had a relatively quiet genome, limited to the 1p and 19q co-deletions. Additional genomic changes were observed in the 1p19q cohort, the majority of which were relatively large scale, tending to encompass entire chromosomes. Genomic gains included trisomy 11, 17 & 21, trisomy 22 and one case that presented a mixed gain of chromosome 7, with 3 copies of 7p and 4 copies of 7q. Genomic losses included monosomy 14, monosomy 15 & 18, and a second case of monosomy 18 (Fig. 1b). These changes occurred case by case and not across the entire cohort, suggesting that additional genomic changes are not specifically associated with the 1p19q co-deletions.

### EGFR Amplified

**Fig. 2:**
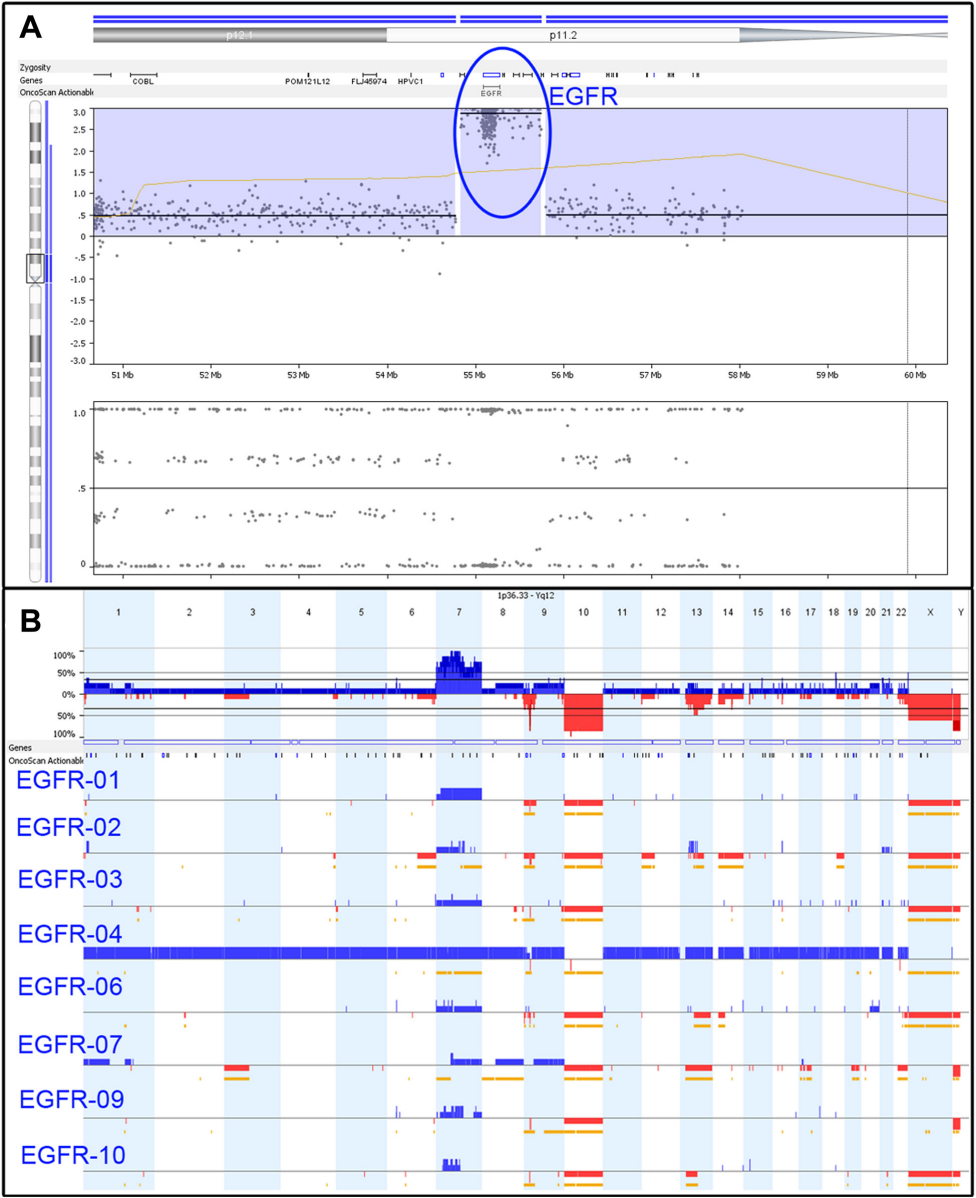
Copy number changes observed in GBMs. **a** A representative sample of EGFR amplification that was confirmed by array in each case (blue circle). **b** Additional CNCs observed across the GBM cohort

**Fig. 3:**
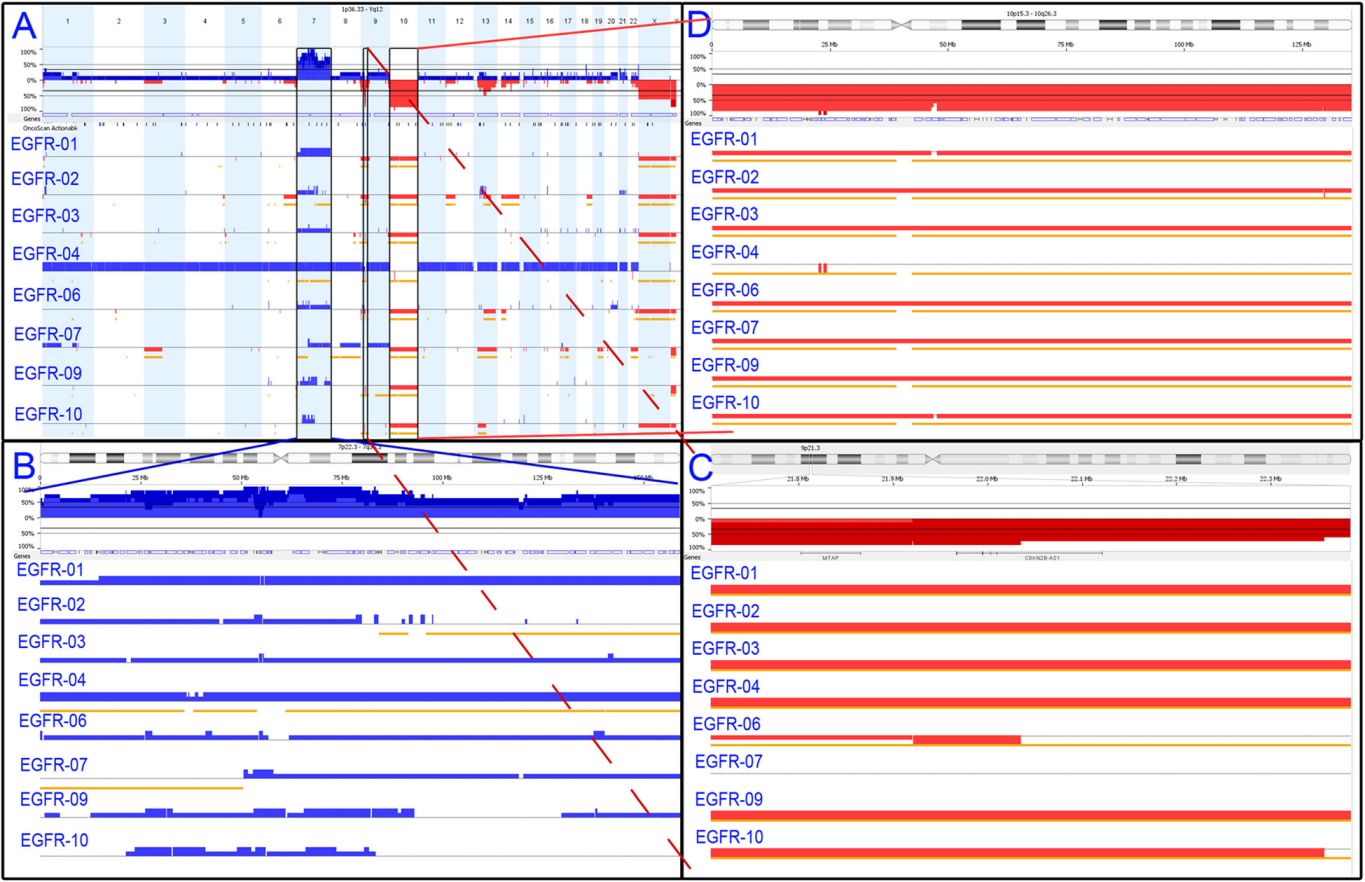
Additional copy number changes observed in the GBM cohort. **a** Whole genome overview of the glioblastoma cohort includes **b** Chromosome 7 complexity, **c** CDKN2A/B deletions and **d** Chromosome 10 deletions

The second cohort contained 8 GBM cases positive for EGFR amplification by FISH. Amplification of EGFR was confirmed in each case by OncoScan (Figs. 2a and 3a). Amplifications varied, ranging from 8–61 copies of the EGFR gene. EGFR copy number (CN) was called by the analysis software, which is designed to estimate the CN based on both the log2 ratio, as well as the estimated tumor content, giving an integer CN call for the tumor portion of the sample in most cases. In some cases, with significant tumor heterogeneity, the tumor content cannot accurately be determined by the software. In these cases the CN calls are binned and portrayed as an average CN change across the entire cell population.

We compared the amplification calls made by FISH with those made by array (Table 1). In addition, 3 samples were repeated on the array to determine reproducibility. FISH copy numbers were calculated as (signals counted)/(number of cells). Similar patterns were observed with respect to relative amounts of amplification. There was some variability in CN estimation—shown by the repeated arrays. However, the advantage of the CN call made by the array is that it is an estimation based on the entire sample, rather than subjectively called after assessing only regions of amplification. For example, EGFR-03 contained patches of high amplification amidst large regions of little or no amplification, within the tumor. The FISH report reflects a focus on scoring amplified regions while passing over “normal” regions. The array data, on the other hand, accounts for both, giving an overall count of EGFR amplification across the tumor. FISH analysis is open to more subjectivity and in cases of high copy gains where signals are clustered together it is often difficult to get an accurate signal count.

**Table 1:** CN comparison of EGFR by array and FISH

Sample	MIP array		FISH
	EGFR CN	Repeat	EGFR CN
EGFR-01	37		>20
EGFR-02	51	37	>10
EGFR-03	8		>13.20
EGFR-04	61		>20
EGFR-06	47		>15.98
EGFR-07	13.33^a^		6.00
EGFR-09	34	27	>16.0
EGFR-10	22	18	16.95

^a^The tumor content could not be determined, likely due to the presence of more than 2 cell populations with differing degrees of CNCs. The CN reported here is therefore an average of the EGFR CN across the entire tissue biopsy

After confirming EGFR amplification we analyzed each case to identify other CNCs found across this cohort. Recurrent CNCs identified in this group included complex gains across chromosome 7, CDKN2A/B deletions, loss of chromosome 10 and chromosomal changes of RB1 affecting 5 of 8 cases (Fig. 2b).

In addition to EGFR amplification we observed a high degree of complex genomic changes involving chromosome 7 (Fig. 3b). All cases within this cohort had gains encompassing at least 39 % of chromosome 7. Complex changes included normal diploid segments, long stretches of single or double copy gains, and isolated regions of amplification not limited to EGFR. It is unclear whether EGFR amplification is a result of overall destabilization of the entire chromosome 7, or rather the initiating cause of it.

Seven cases (88 %) contained homozygous deletions of CDKN2A/B (Fig. 3c), which is commonly associated with EGFR amplification in GBM [6, 7]. The last case (EGFR-07) had a normal diploid count along the 9p arm, including CDNK2A/B. The remainder of chromosome 9 presented a mosaic gain, indicating a relative loss of 9p (CDNK2A/B), although not to the same extent as the other cases. EGFR-07 was one of 2 cases that also contained a complete deletion of the RB1 gene locus. Disruption of the Rb pathway is a primary component of glioma development and can occur through loss of RB1 or CDKN2A/B expression or through amplification of CDK4 [8, 9, 7]. It is possible that, in this case, deletion of the RB1 gene precluded the need for CDKN2A/B deletion. In addition to CDNK2A/B, the methylthioadenosine phosphorylase (MTAP) gene also falls within the deleted region, although in EGFR-06 only a hemizygous loss is observed at the MTAP position as compared to a very focal homozygous deletion of the CDNK2A/B genes. Although MTAP has not been extensively characterized, it is commonly deleted in a number of cancers and there is some evidence that is has tumor suppressive properties [10–12].

The third recurrent CNC observed across this cohort was a chromosome 10 deletion (Fig. 3d), observed in all eight cases (100 %). One case, EGFR-04, presented copy-neutral LOH, apparently due to a hemizygous loss of chromosome 10 followed by genome duplication. Deletion of 10q is commonly reported in GBM [13–16], and may be a mechanism for the inactivation of the tumor suppressor phosphatase and tensin homolog (PTEN), which has been associated with GBM [17]. LOH resulting from 10q deletion may result in haploinsufficient expression, or exposure of inactivating PTEN mutations within the retained allele. However, such a large scale loss may indicate that additional tumor suppressor genes across chromosome 10 may also play a role in retaining cell-cycle equilibrium. Two putative tumor suppressor genes potentially impacted by chromosome 10 LOH are ANXA7 and PFKFB3. ANXA7 acts as a positive regulator of EGFR, and haploinsufficiency of ANXA7 reportedly results in stabilized EGFR protein [18]. Fleischer and colleagues showed that LOH of the PFKFB3 gene results in the reduction of UB12K4 expression, a growth-inhibiting splice variant of PFKFB3. They concluded that this shift in UB12K4 expression tends toward more aggressive tumor growth [19].

The final recurrent CNC observed in this cohort was a deletion overlapping the RB1 gene in 4 of 8 (50 %) cases. RB1 gene inactivation has been identified in numerous cancers [20] either through inactivating mutations or RB1 deletions (reviewed in [7]). Two cases (EGFR-02 & EGFR-06) contained partial deletions of RB1, whereas EGFR-07 & EGFR-10 had deletions spanning the entire gene locus. As mentioned above, EGFR-07 was the only case in which the CDKN2A/B locus was not homozygously deleted, which may be in part a result of the RB1 deletion—causing a prior disruption in the Rb pathway which is often accomplished by the loss of the CDKN2A/B gene locus. CDKN2A expression has therapeutic implications, which in turn may be blocked by the loss of RB1. For example, deletion of CDKN2A is a marker of increased sensitivity to the CDK4/6 inhibitor PD0332991 in melanoma. However, RB1 deletion leads to PD0332991 resistance, counteracting the CDKN2A-imbued sensitivity [21]. Testing in GBM cell lines and GBM xenografts have shown similar results, indicating that PD0332991 could be effective in treating gliomas, which typically have CDKN2A/B deletions. Similarly, PD0332991 resistance was exhibited by cell lines with RB1 deletion, and also by cell lines lacking the expression of functional Rb1 protein [22].

## Conclusions

Microarray enables us to gain a broader picture of the CNCs occurring in glioma cases. Gliomas with 1p19q co-deletion have a relatively quiet genome, apart from the obvious co-deletion. Additional CNCs are observed in isolated cases, but appear to occur primarily as larger aberrations—such as complete loss or gain of entire chromosomes. On the other hand, EGFR amplified cases tend to be more complex and have specific abnormalities associated with the EGFR amplification. Furthermore, 1p19q co-deletions and EGFR amplification associated CNCs appear to be mutually exclusive.

## Methods

The samples used in this study were de-identified and the study was conducted under the University of Utah Institutional Review Board protocol 7275. Seventeen cases were selected retrospectively from confirmed glioma diagnoses based on positive FISH results for either 1p19q co-deletions (n = 9) or EGFR amplification (n = 8). All 17 cases were processed on the OncoScan array designed for copy number analysis of FFPE samples. Briefly, the OncoScan platform uses MIP probes—linear probes containing 2 genomic homology regions separated by a linker DNA. The probe forms an inverted loop and binds the gDNA with the homology regions next to each other flanking a SNP. A dinucleotide complementary to the SNP is incorporated into the probe resulting in circularization of the MIP probe, then the gDNA template and unutilized probe are removed via exonuclease degradation of linear DNA. The probe is cleaved between universal primer sequences encoded in the linker region of DNA, which allows for subsequent amplification of the probe. In the context of the OncoScan array, the linker region also encodes a unique molecular tag corresponding to the genomic region encoded in the probe. This unique tag is then separated from the probe and used to interrogate the array.

The raw data was processed using OncoScan® Console (Affymetrix, Santa Clara, CA) and all cases were analyzed with Nexus Express software (BioDiscovery, Hawthorne, CA) to assess CNCs across the genome and identify consistent patterns across each class of gliomas. All calls made by the software were manually reviewed for probe performance.

## Abbreviations

FFPE: Formalin-fixed, paraffin-embedded; SNP: Single nucleotide polymorphism; GBM: Glioblastoma
CN: Copy number
CNC: Copy number change
LOH: Loss of heterozygosity
CN-LOH: Copy neutral-loss of heterozygosity
FISH: Fluorescent in situ hybridization
WHO: World Health Organization
